# Clinical Team Response to the Impact of COVID-19 on Diabetes Self-Management: Findings From a Qualitative Study

**DOI:** 10.3389/fcdhc.2022.835845

**Published:** 2022-03-10

**Authors:** Lily Hale, Thomas C. Cameron, Katrina E. Donahue, Maihan B. Vu, Jennifer Leeman, Asia Johnson, Erica Richman, Jennifer Rees, Laura Young

**Affiliations:** ^1^ School of Medicine, University of North Carolina at Chapel Hill, Chapel Hill, NC, United States; ^2^ Department of Medicine, Division of Endocrinology and Metabolism, University of North Carolina at Chapel Hill, Chapel Hill, NC, United States; ^3^ Department of Family Medicine, University of North Carolina at Chapel Hill, Chapel Hill, NC, United States; ^4^ Cecil G. Sheps Center for Health Services Research, University of North Carolina at Chapel Hill, Chapel Hill, NC, United States; ^5^ Department of Health Behavior, Gillings School of Global Public Health, University of North Carolina at Chapel Hill, Chapel Hill, NC, United States; ^6^ School of Nursing, University of North Carolina at Chapel Hill, Chapel Hill, NC, United States; ^7^ North Carolina Translational and Clinical Sciences Institute, University of North Carolina at Chapel Hill, Chapel Hill, NC, United States

**Keywords:** diabetes, self-management, self-care, COVID-19, qualitative research, mental health

## Abstract

The aims of this study were to explore providers’ perceptions of how COVID-19 affected patients’ psychological wellbeing and diabetes self-care and discover how providers responded to sustain and improve patients’ psychological health and diabetes management during the pandemic. Twenty-four semi-structured interviews were completed with primary care providers (n=14) and endocrine specialty clinicians (n=10) across sixteen clinics in North Carolina. Interview topics included: (1) current glucose monitoring approaches and diabetes management strategies for people with diabetes (2) barriers and unintended consequences encountered with respect to diabetes self-management, and (3) innovative strategies developed to overcome barriers. Interview transcripts were coded using qualitative analysis software and analyzed to identify cross-cutting themes and differences between participants. Primary care providers and endocrine specialty clinicians reported that people with diabetes experienced increased mental health symptoms, increased financial challenges and positive and negative changes in self-care routines due to COVID-19. To offer support, primary care providers and endocrine specialty providers focused discussions on lifestyle management and utilized telemedicine to connect with patients. Additionally, endocrine specialty clinicians helped patients access financial assistance programs. Findings indicate that people with diabetes experienced unique challenges to self-management during the pandemic and providers responded with targeted support strategies. Future research should explore the effectiveness of these provider interventions as the pandemic continues to evolve.

## Introduction

COVID-19 restrictions challenged patient-centered care for diabetes. Early in the pandemic, many ambulatory practices halted their in-person visits in favor of virtual appointments (1–3). This posed a problem for the management of diabetes, which typically requires routine in-office monitoring, lab testing, and medication management. In the first two months of the pandemic, rates of HbA1c tests fell by as much as 66% (4). There was concern among the diabetes healthcare community that the pandemic would result in poor diabetes outcomes, as interruptions in appointments and HbA1c testing have previously been associated with worsened glycemic control (5, 6).

In addition, people with diabetes have struggled with self-management practices in the face of COVID-19. People with diabetes report eating more and exercising less compared with prior to the pandemic (7–9). Members of the diabetes community also report concerns over finances and employment, which may have impacted insurance status and access to care (8). Additionally, disruptions in diabetes supplies have affected individuals’ ability to adhere to medication and self-monitoring recommendations. A U.S. survey of people with diabetes found that one in six people needing insulin experienced a problem. A similar proportion of people had issues obtaining test strips, and 25% had difficulties obtaining pumps or continuous glucose monitoring supplies (10).

In the face of these significant challenges, it is unsurprising that people with diabetes report higher levels of stress and depression than prior to the pandemic (10–12). Restrictions on gatherings limit individuals’ access to social support systems and may exacerbate these mental health effects (13). Stress and depression affect glycemic control directly and through their impact on diabetes self-care (14). Therefore, it is imperative for providers to recognize the diverse array of challenges COVID-19 presents to their patients with diabetes and develop targeted strategies for support. In this study, we (1) explore providers’ perceptions of how COVID-19 affected patients’ psychological wellbeing and diabetes self-care and (2) identify how providers responded to sustain and improve patients’ psychological health and diabetes management during the pandemic, with a focus on self-monitoring of blood glucose, A1c testing, dietary intake, and physical activity.

## Methods

### Study Design

The study used a qualitative design to collect data through in-person and telephone interviews. These in-depth, semi-structured interviews provided rich and detailed descriptions regarding provider impressions of challenges facing patients with diabetes during COVID-19. This study was conducted in the context of a larger study, Re-Think the Strip (RTS), which aims to promote the de‐adoption of daily self‐monitoring of blood glucose (SMBG) among non‐insulin treated patients with controlled type 2 diabetes given the lower utility of this practice (15–18). Rethink the Strip involves 20 primary care clinics in the North Carolina. We were concerned that decreases in access to A1c test results would lead patients and providers to question the wisdom of de‐adopting SMBG during the pandemic. To address this concern, we conducted interviews with staff members within the existing RTS clinics and expanded the interviews to endocrinologists in North Carolina to obtain a more complete picture of diabetes care during COVID-19. Primary care providers focused their discussion on patients with type 2 diabetes, while endocrinologists commented on their experience treating patients with type 1 and type 2 diabetes.

### Study Population

Our study population comprised of 24 diabetes clinicians and staff. This included 9 primary care clinicians and 5 primary care ancillary staff members from the existing RTS practices, and 10 endocrine specialty clinicians. Participants are summarized in **Table 1**. Participants were identified and recruited using purposive sampling based on their ability to provide in-depth, detailed information about diabetes care during COVID-19.

**Table 1:** Interview participants.

Participant Role, Title	Number
**Medical Director**	1
**Primary Care Physician (MD)**	2
**Nurse Practitioner (NP)**	1
**Practice Manager**	1
**Physician Assistant (PA)**	5
**Registered Dietician (RD)**	3
**Registered Nurse (RN)**	1
**Endocrinologist (MD)**	10
**Total**	24

### Data Collection

Semi-structured, in-depth telephone and in-person interviews were conducted from September 2020-August 2021. Before each interview, the researcher explained the goals of the study, reviewed confidentiality measures, and obtained verbal consent. All interviews were recorded using a field recorder with participant consent. The interviews were conducted in English. An interview guide (**Table 2**) was developed prior to participant interviews, based on literature review and previous work completed by the RTS team. Participants were asked a series of pre-determined open-ended questions and follow-up probes were generated based on participant responses. Each interview lasted approximately 35 minutes.

**Table 2:** Key interview questions.

Dimensions	Open Ended Questions
**Glucose Monitoring Approaches**	As a health care provider, what have been the biggest changes in terms of patient care? How is your practice currently addressing A1c monitoring? How has your recommendation on glucose monitoring changes, if at all, since COVID-19?
**Changes and Barriers in Patients Self-Management Practices**	What concerns have your patients shared about their diabetes care during COVID-19? How has COVID-19 impacted your patients’ ability to adhere to your self-management recommendations? What are challenges your patients have shared about their new glucose monitoring approach or self-management?
**COVID-19 Health and Outcomes**	To get an overall sense of diabetes control, how often do other indicators like diet, weight, or blood pressure enter the discussions? During this time of COVID-19, for your patients with diabetes, what additional health issues or conditions have come to your attention? Since COVID-19, what changes, if any, have you noticed with your patients’ glycemic control?

### Data Analysis

Interviews were professionally transcribed and reviewed by a team member for accuracy. Interview transcripts were independently analyzed by two investigators using content analysis. A codebook was developed by the research team, consisting of *a priori* codes derived from discussion questions and additional concepts that emerged from analysis. ATLAS.ti 9, a qualitative software program, was used to facilitate the analysis (19). Differences and discrepancies were discussed and reconciled. Cross-cutting themes and differences between participants were identified.

## Results

Participant impressions of the impact of COVID-19 on patient self-management were categorized into five key themes, summarized in **Table 3**. Illustrative quotations from primary care providers and endocrine specialty clinicians are presented to highlight study findings.

**Table 3:** COVID-19 impacts on self-management.

Theme	Subtheme and Illustrative Quotation
**Increase in mental health symptoms made self-care difficult.**	**Depression** *Then there’s been quite a bit of depression too. Not necessarily depression that needs medication, but definitely some decreased moods just with patients not being able to see their loved ones, or see their friends, or get out and do anything. (Primary Care Provider)*
	**Anxiety** *We’ve seen more anxiety, a lot more anxiety, a lot more anxiety and depression as well. But I think it’s been, I would say, maybe 75/25 anxiety/depression for me because I only see women. I think women, especially women with young children or families, they’re taking this very seriously some of them. It’s really affecting them mentally because they’re the protectors of the children, and they can’t protect their children, and it’s really working their nerves very badly. (Primary Care Provider)*
	**General stress and wellbeing** *In general I think more folks were just feeling down. In general you could feel that they were stressed. A lot of their normal routine was disrupted, and they were not able to keep up with their families just because everyone was somewhat staying at home. So, I think it was definitely a time where people were just not feeling well. (Endocrine Specialty Clinician)*
**Financial challenges impacted self-care.**	**Job or insurance loss** *I think they had more challenges because a lot of people did lose jobs and it was a big challenge to go to doctors and paying copays and everything. They lost insurances too. That way I think they’re also having issues paying bills for their medical health. (Endocrine Specialty Clinician)*
	**New Expenses** *People who are not working their full hours, people who were having to pay for childcare that didn’t usually because the kids were in school … I think a lot of people were having to pinch a penny here or there, and if they weren’t really vested in doing [self-management] before, it was certainly one of the easier things to cut loose. (Primary Care Provider)*
**Restrictions disrupted self-care routines.**	**New family responsibilities** *The people who suffer the most are the same people who always suffer the most, women for sure, childcare. There was always kids in the background. They’re the ones for the most part stuck with childcare, and elder care, and everything else. (Endocrine Specialty Clinician)*
	**Reduced safe exercise space** *They’re not able to go to the gym for a long time … Some of them are even afraid to go out and walk which I kind of told them that hey, you can go outdoors without a mask if you’re not around other people it’s okay but some were not willing to do that initially so many patients have gained weight. (Endocrine Specialty Clinician)*
	**Reduced access to healthy food** *Of course, a lot of people are stress eating or tending to eat food because they really don’t want to go to the grocery store as often, so they’re not buying as many fresh fruits and vegetables. They’re tending to eat more processed, non-perishable foods. (Primary Care Provider)*
	**Increased availability of unhealthy food** *Their access to food ended up being worse because they were doing drive-thru rather than going to the grocery store. (Endocrine Specialty Clinician)*
**Restrictions allowed patients to prioritize self-care.**	**Improved diet** *They would say, “Yeah, it was great to be home with the kids, and I also cooked all the time, so I did well with my blood sugar monitoring and carb intake,” (Endocrine Specialty Clinician)*
	**Increased exercise** *I have occasional patients who now find themselves with more free time because of COVID. Maybe their work hours have been cut back or whatever, so they’re actually exercising more. (Primary Care Provider)*
	**More time for self-management** *A lot of my patients cut down on one to two hours of commuting a day into their office … they were able to check for the first time three times a day before they ate their meals and could decide how much insulin to give which, in an office environment, sometimes, they wouldn’t check before lunch because they didn’t want to do it or didn’t have time. (Primary Care Provider)*
	**Improved participation in care** *And that actually made my patients a little bit more participatory in their diabetes care because there was all these other setbacks meaning they couldn’t come in for routine bloodwork and evaluation in a clinic type setting. (Endocrine Specialty Clinician)*
**Clinical team utilized patient-centered strategies.**	**Prioritized lifestyle discussions** *With the gyms closing and now that they’re back open some people do feel comfortable to go back but some people are still saying “yeah, I’m still not feeling great about it” but then kind of having the discussion of “alright, I get it, I’m not going back to the gym either right now but it is still nice outside and you can walk” and then they’re like “oh yeah, I guess I could do that” … so having to make more of a conscious effort to go for a walk, to get up and move every hour or so to get some more steps in. (Primary Care Provider)*
	**Utilized telemedicine** *I would say in the patient who is pretty tech savvy and can upload their CGM or their pump, the virtual visits actually work quite well because we have all of the data in front of us so especially if they’re on a CGM, we can estimate their A1C just from that. In that population, I think it actually went pretty well. (Endocrine Specialty Clinician)*
	**Offered financial assistance** *We encouraged patients to apply for drug assistance, and sometimes helping them out with doing that. We directed them to clinics, pharmacies, clinics that have pharmacies for indigent patients that offer a drug program that they can access. All of that, and then we do have an active sampling system (Endocrine Specialty Clinician)*

### Theme 1: Providers Observed an Increase in Mental Health Symptoms Which Made Patient Self-Care More Difficult

Primary care and endocrine providers reported patients had increased mental health symptoms, mainly depression, anxiety, and general stress. These symptoms presented challenges to diabetes self-management.

#### Depression

Both primary care providers and endocrine specialty clinicians noticed that more patients were struggling with depression compared with time prior to the pandemic. Some patients’ depression stemmed from isolation and lack of social support due to COVID-19 restrictions. One endocrine provider stated:

*I would say definitely mental health has suffered in all my patients including diabetes patients, lots of just social isolation and general isolation. I’m definitely seeing more depressive symptoms in that regard… *

A primary care provider echoed these sentiments:

*Then there’s been quite a bit of depression too. Not necessarily depression that needs medication, but definitely some decreased moods just with patients not being able to see their loved ones, or see their friends, or get out and do anything.*

Isolation seemed to be especially difficult for patients with diabetes who had incorporated social support groups into their self-care routines.

*I think there was a component of maybe even like loneliness or what is it called, like the diabetes distress … I had an older population that did group classes and things like that. They didn’t have the gym to go out and socialize with, which caused them to be depressed, which would cause them to not take as good a care of themselves as they could. Patients that did walking groups, things like that, again, they didn’t have that support. (Endocrine Specialty Clinician)*

Patients also experienced depression following the loss of family and friends during the pandemic.

*Depression is very common, stress and they have lost a lot of loved ones and their family which led to stress and stress eating which led to everything like the weight gain and poor control. (Endocrine Specialty Clinician)*

#### Anxiety

Both endocrine specialty clinicians and primary care providers endorsed that patients experienced increased rates of anxiety during the pandemic. One source of anxiety was the risk of contracting COVID-19, especially given the high-risk status of people with diabetes.

*I’m definitely seeing more depressive symptoms in that regard, more anxiety as there was worry about contracting the virus and worry about going out in public, so I’ve definitely seen that. (Endocrine Specialty Clinician)*

Another significant source of anxiety was financial concerns, as patients lost their job and were forced to juggle competing priorities.

*People are suddenly having to feel the financial losses and that sort of thing with COVID. I think people are definitely stress eating more or just distracted from their own health issues because they suddenly have – trying to figure out how to find another job or how they’re going to pay their rent. Dealing with their diabetes is sort of a lower thing on their priority list. (Primary Care Provider)*

Finally, participants reported that caretakers experienced increased anxiety over the desire to protect their loved ones during the pandemic.

*We’ve seen more anxiety, a lot more anxiety, a lot more anxiety and depression as well. But I think it’s been, I would say, maybe 75/25 anxiety/depression for me because I only see women. I think women, especially women with young children or families, they’re taking this very seriously some of them. It’s really affecting them mentally because they’re the protectors of the children, and they can’t protect their children, and it’s really working their nerves very badly. (Primary Care Provider)*

#### General Stress and Well-Being

Primary care providers and endocrine participants noted that patients were also facing more general mental health challenges, such as *“just feeling down”*, or experiencing *“high levels of stress”*. These mood changes resulted in patients becoming increasingly isolated and, for many patients, translated to more difficulty with diabetes-self management practices.

*I see that the stress is impacting their eating, emotional eating. It’s impacting their blood pressure. It’s impacting their food choices which is ending up sometimes causing their cholesterol to go up or their blood sugars to be higher so emotional eating, stress eating and things like that. (Primary Care Provider)*

### Theme 2: Patients Encountered Financial Challenges Which Impacted Self-Care

Participants noted patients had trouble affording diabetes supplies, medications, or healthy food due to job or insurance loss or new expenses.

#### Job or Insurance Loss

Both primary care providers and endocrine specialty clinicians explained that patients had trouble following self-management strategies due to “*financial hardships with some people losing their job”*. As one primary care provider explained, “*patients who have lost their job often times also have lost their health insuranc”.*. Financial pressures forced patients to choose what expenses to prioritize. Diabetes self-management practices “*seem to be easier things to let go of because it’s not life-threatening to not test your blood sugar”*. One endocrine specialty clinician summarized:

*We have several patients that had to start working two jobs because of COVID and a lot of them were laid off. So a lot of patients lost insurance during COVID. So then they stopped their medications and didn’t check their blood sugar because test strips are expensive.*

#### New Expenses

In addition to loss of income, primary care and endocrine participants noted that patients had new expenses, such as childcare.

*People who are not working their full hours, people who were having to pay for childcare that didn’t usually because the kids were in school … I think a lot of people were having to pinch a penny here or there, and if they weren’t really invested in doing [self-management] before, it was certainly one of the easier things to cut loose. (Primary Care Provider)*

*The cost of the medication in diabetes care has been an issue even as I said pre-pandemic and then the pandemic put more stress on it. People lost their jobs and things like that or they had some other expenses. Let’s say child care for example and things like that so the cost of diabetes medication I would say is probably … challenging. (Endocrine Specialty Provider)*

### Theme 3: Restrictions Created Disruptions to Patient Self-Care Routines

Participants discussed the ways that COVID-19 restrictions disrupted patient self-care routines, including new family responsibilities and changes to diet and exercise habits.

#### New Family Responsibilities

Endocrine specialty clinicians noted that new *“childcare”* or “*eldercare”* responsibilities impacted patients’ ability to practice self-care. One clinician noted that for many patients, especially women, there were *“always kids in the background”* during virtual appointments.

*People with diabetes that were having distress related to care that had children at home and then were doing the homeschooling and things like that, their control definitely deteriorated because they were struggling to manage just day-to-day activities… (Endocrine Specialty Clinician)*

Another endocrine clinician wondered if family responsibilities may have played a role in the differences he observed between type 1 and type 2 diabetes patients:

*Actually, come to think of it, the majority of my type 1’s would be in their 30’s or maybe 40’s … And with the pandemic, schools were shut so everything was virtual. So now I can imagine if they had to help with their kids as well as thinking of themselves, that would have been a lot more challenging. With my type 2’s, a little bit older in general, and so their kids are already grown and that’s not a concern. So, maybe they had more me time. (Endocrine Specialty Clinician)*

#### Reduced Safe Exercise Space

Endocrine and primary care providers discussed barriers to safe exercise during COVID-19. Gym closures disrupted patients’ exercise routine, and it was difficult to find a safe alternative.

*We have to talk a lot about weight and exercise even when the gym is closed or even when they don’t feel comfortable going to the gym even when it’s re- opened and when they’re trying to really socially distance, and so they don’t want to walk in the mall anymore, and that sort of thing. Of course, they all, by definition, are high risk if they got COVID, so it’s even trickier with that population to find ways that they can safely exercise. (Primary Care Provider)*

For people with diabetes, their high-risk status created significant distress about leaving the house to exercise. One endocrine provider noted that some patients were “*afraid to go out and walk”*. A primary care provider with a similar experience said, *“they feel like they have to wear a mask outside because somebody might walk too close to them and they can’t breathe with the mask outside because it was too hot”*.

Additionally, working from home reduced patients’ level of activity.

*I’ve said multiple times to people, “You’re not working. You’re working from home. You’re sitting in front of a computer eight hours a day”. I have a teacher who she just absolutely couldn’t understand why she had gained weight, and I said, “You’re not chasing around a five-year-old anymore for eight hours a day”, and so we’ve seen a lot of that. (Primary Care Provider)*

*People did not walk. People didn’t go out to play or to exercise as much as they would. People didn’t go to the gym as much as they did. The lifestyle management aspect of diabetes management went out the window. (Endocrine Specialty Clinician)*

#### Reduced Access to Healthy Food

Primary care and endocrine providers noted that concerns over COVID-19 impacted patients’ access to healthy food, because “*These patients don’t want to get out and go to the grocery store, go to the farmers market or whatever”.* Because patients were hesitant to make frequent trips to the grocery store, “*they’re not buying as many fresh fruits and vegetables”.* If patients did make it to the grocery store, healthy options were not always available.

*I also heard patients saying that they were eating what was available in the supermarkets and that they couldn’t always find what it is that they felt they should be eating, and they had to just take what was there at one point. (Primary Care Provider)*

#### Increased Availability of Unhealthy Food

Primary care and endocrine providers discussed the increased accessibility of unhealthy food during the pandemic. An endocrine provider described that for some patients, “*their access to food ended up being worse because they were doing drive-thru rather than going to the grocery store”.* One primary care provider explained that if patients went to the grocery store, they tended to buy “*more processed, non-perishable foods*” to reduce the frequency of grocery trips. Additionally, a primary care provider explained that lockdowns and work from home placed many patients “*within twenty feet of their refrigerator”,* with constant access to less healthy foods.

Primary care and endocrine providers noted that this increased availability coupled with emotional eating led to an increase in unhealthy food consumption. Patients turned to food to deal with *“stress”, “boredom”* or to find *“comfort”.* As one primary care provider summarized, *“People working at home and having easy access to snacks and being stressed and stress eating”* often resulted in weight gain.

### Theme 4: COVID-19 Restrictions Allowed Patients to Prioritize Self-Care

More endocrine providers than primary care providers noted that for some patients, lockdowns allowed more time for self-management, encouraged healthier routines, and improved patient participation in care.

#### Improved Diet

Endocrine and primary care providers noticed that lockdowns and working from home allowed patients to cook meals at home and pay more attention to their diet. One primary care provider stated that for many patients, eating out at restaurants *“wasn’t an option, many more people were cooking more so I have noticed a change over time where a lot of people I was talking to were cooking more often, eating out less”.* An endocrine provider with a similar experience recalled patients saying, *“‘Yeah, it was great to be home with the kids, and I also cooked all the time, so I did well with my blood sugar monitoring and carb intake’”.*

One endocrine provider noticed an improvement in patients with type 2 diabetes:

*I think it has gotten better for my type 2 because they were able to appreciate their caloric intake, and they were actually prepping the meals themselves. They no longer had access to eating at restaurants, which was the majority, 70 percent of their situation prior to Covid. And so I think it helps with betterment of glycemic control during the pandemic in the shutdown.*

#### Increased Exercise

Endocrine and primary care providers felt that for some patients, working from home led to increased opportunism for exercise. One endocrine provider stated, *“a lot of my patients cut down on one to two hours of commuting time a day”* which allowed them to “*walk more”* for exercise. A primary care provider observed how job loss allowed more time for exercise:

*I have occasional patients who now find themselves with more free time because of COVID. Maybe their work hours have been cut back or whatever, so they’re actually exercising more. I have a couple of patients who have actually lost a lot of weight during COVID.*

#### More Time for Self-Management

Endocrine and primary care providers discussed that some patients’ self-management improved because of *“more free time”.* An endocrine provider noted that patients “*weren’t obligated to go somewhere to partake in or help out in other scenarios”* so they *“had more time to themselves”.* One endocrine provider described how working from home made monitoring blood sugar more convenient:

*They were able to check for the first time three times a day before they ate their meals and could decide how much insulin to give which, in an office environment, sometimes, they wouldn’t check before lunch because they didn’t want to do it or didn’t have time, and they got home after dinner, and they were eating on the road, so they didn’t have time to check their sugar or give their insulin. For a small subset, maybe not small, but for a subset, I would say they increased their blood sugar monitoring because they had more time, and they were able to take better care of themselves.*

#### Improved Participation in Care

Endocrine providers discussed how aspects of the pandemic made patients *“more participatory in their diabetes care”.* One endocrine provider noted that because patients *“couldn’t come in for routine bloodwork and evaluation in a clinic type setting”,* they were more motivated to care for their diabetes. Another endocrine provider explained that the risk of COVID-19 encouraged more participation in care:

*In general, COVID was a very scary event for them. And so they tried really hard to get their sugars under control because they were very afraid of winding up in the hospital and getting very ill. And because there were fewer no-shows … because we did telemedicine they were more engaged. And our CDEs were excellent in having visits between the physician visits. And so overall, I think, after we all got through the craziness of COVID, we felt more connected. And patients felt more engaged, more a sense of responsibility over caring for themselves … And it’s not just a theoretical risk of harm if you don’t take your medications, but it was a very real risk. So I would say that there’s a lot of education that was achieved during COVID and then more ownership of their care.*

### Theme 5: Clinical Team Members Utilized Patient-Centered Management Strategies

Primary care and endocrine providers discussed the strategies used to help patients maintain optimal self-care during the pandemic, including prioritizing lifestyle discussions, utilizing telemedicine, and helping with financial assistance.

#### Prioritizing Lifestyle Discussions

Primary care and endocrine providers made lifestyle discussions a priority during COVID-19. Discussions with patients focused on creative ways to stay active despite pandemic restrictions.

*With the gyms closing and now that they’re back open some people do feel comfortable to go back but some people are still saying “yeah, I’m still not feeling great about it” but then kind of having the discussion of “alright, I get it, I’m not going back to the gym either right now but it is still nice outside and you can walk” and then they’re like “oh yeah, I guess I could do that” … so having to make more of a conscious effort to go for a walk, to get up and move every hour or so to get some more steps in. (Primary Care Provider)*

*When it came to weight, I tried to coach as much as possible because a lot of the gyms were shut down during COVID. What I would do is encourage them to go for short walks after the biggest meal of the day to help keep blood sugars under control as well as cholesterol. I encouraged them to start out at like just five minutes at a time and then build up to 30 minutes. That was probably the most common instructions I gave for weight management and weight control. (Endocrine Specialty Clinician)*

#### Utilizing Telemedicine

Primary care and endocrine providers noted that the use of telemedicine had allowed them to stay connected to their patients during COVID-19. Although the physical exam was limited and accessing blood glucose data was challenging, providers felt that it was largely successful.

*I don’t think I’ve had anybody where we’ve kind of not been able to accomplish what we kind of want to get done [with a virtual visit]. Obviously we can’t do like foot exams and things like that. Obviously there are some cases where we want to know what the A1C is or we want to know what the blood sugar is and we can’t check it but I think in those cases where we’re kind of saying alright well let’s see how things are looking in another couple of months and we can do it then. But I think overall it’s been pretty good. I’ve been able to accomplish all I want to accomplish pretty much. (Primary Care Provider)*

Endocrine participants noted that telemedicine visits were especially successful when patients were able to share glucose data through CGM devices or pumps.

*Yeah, my hope is that there will be a continued option for virtual because I do think it was helpful and worked well in certain circumstances. Yeah, I would hope that we would still have that as an option. Diabetes specifically, I would say in the patient who is pretty tech savvy and can upload their CGM or their pump, the virtual visits actually work quite well because we have all of the data in front of us so especially if they’re on a CGM, we can estimate their A1C just from that. In that population, I think it actually went pretty well. (Endocrine Specialty Clinician)*

Endocrine providers felt telemedicine offered patients a convenient way to access their healthcare team. This seemed especially beneficial for patients in rural areas, or who were busy with work or family obligations.

*Now, it’s been great for patients that live very far away because I’m the only endocrinologist in this part or in this county that takes Medicare/Medicaid, so I have a lot of patients that travel greater than an hour to see me. They don’t necessarily have reliable transportation unfortunately, so being available by telehealth has allowed them to have their diabetes monitored more routinely. (Endocrine Specialty Clinician)*

*We have several patients that had to start working two jobs because of COVID and a lot of them were laid off. So they were doing you know a lot of temp jobs. And so they had no time to come into clinic. So when I called them for a tele-visit, it was almost always at work. And they were in their car or driving an Uber, for example, they would pull over and will conduct the visit. So we had extremely high satisfaction rates because we were able to offer tele medicines, you know, even up till now. And so actually our no-show rate was the lowest it’s been because people didn’t have to take public transportation in and they didn’t have to take out like a whole half day from work. (Endocrine Specialty Clinician)*

Endocrine participants did note that the benefits of telemedicine were limited for older patients who had difficulty interfacing with the technology and for patients without reliable access to a computer or internet.

*So there’s definitely barriers when a patient doesn’t have access to technology, so if they don’t have a smartphone, or a computer, then you’re severely limited. And so it doesn’t work as well … But so I would say, you know, and that’s sad, because the patients that are economically disadvantaged, still have barriers to care, because this is mostly technology based. (Endocrine Specialty Clinician)*

*There just seems to be kind of an age barrier to the virtual as far as especially over 65 for a diabetes visit where they have to figure out how to upload [CGM] or something. I think that just becomes a significant burden. And sometimes even just trying to figure out how to get my microphone on or my camera on in that age group is just a big source of stress on their part. And they will be begging to come in even if we advise them not to. (Endocrine Specialty Clinician)*

#### Offering Financial Assistance

Endocrine providers aimed to help patients afford diabetes supplies and medications by suggesting inexpensive options, offering product samples or helping patients apply for drug assistance programs.

*So I tell them to go to Walmart and buy a Walmart meter which I find that out of all the generic, cheap meters out there out there it’s probably more reliable than some of them and are more affordable and they can get it at a much cheaper price and the test strips are also a lot cheaper so I’m trying to encourage them to not worry about the insurance and get more testing supplies from Walmart. (Endocrine Specialty Clinician)*

*We encouraged patients to apply for drug assistance, and sometimes helping them out with doing that. We directed them to clinics, pharmacies, clinics that have pharmacies for indigent patients that offer a drug program that they can access. All of that, and then we do have an active sampling system (Endocrine Specialty Clinician)*

## Discussion

The COVID-19 pandemic caused significant impacts on the lives of people with diabetes. Based on this qualitative study, primary care and endocrine providers noted that people with diabetes had increases in mental health symptoms, increased financial challenges and positive and negative changes in self-care routines. Primary care and endocrine providers discussed prioritizing lifestyle discussions and noted more use of telemedicine, specifically in blood glucose review. Results from this study can help guide management of people with diabetes as the pandemic evolves.

Our study found that most primary care and endocrine providers noted an increase in mental health concerns in their patients with diabetes. This included a subjective increase in the number of patients complaining of depression, anxiety and stress. These symptoms stemmed in part from social isolation and concern over the high risk of severe infection in the diabetes population. Diabetes was already associated with an increase in mental health conditions prior to the pandemic, and early in the pandemic concerns existed regarding worsening mental health during the pandemic in patients with diabetes (20). A recent study found that 93% of patients with diabetes showed signs of mental suffering and 43% had signs of severe distress (12). Additionally, one in 10 patients with diabetes had suicidal thoughts during the pandemic (21).

Primary care and endocrine providers noted disruptions in self-care in patients with diabetes during the pandemic. A significant theme was a decrease in physical activity and an increase in unhealthy eating patterns. People with diabetes struggled to find safe ways to exercise and obtain healthy foods while protecting themselves from the virus. However, a minority of providers reported patients had improved diets, exercise and time for self-management with a new change in routine. A recent qualitative study investigating the impact of the pandemic on Chinese patients with diabetes found decreases in physical activity, increased anxiety and feeling of lack of support from healthcare professionals (22). However, distinct differences in this study included complaints of lack of access to blood glucose monitoring and lack of space to perform physical activity related to strict quarantine/isolation procedures. An additional study looking at the effect of COVID-19 on self-management in patients with type 2 diabetes using a DMSQ assessment found decreases in physical activity and decreased use of health services (23). Therefore, the literature seems to support the negative changes in self-care routines most of our providers described.

An important question is how these changes in self-care ultimately effect glycemic control. Despite early concerns regarding glycemic control, numerous studies during the pandemic have shown no change in diabetes control in patients with diabetes (24, 25). Additionally, a recent meta-analysis noted a modest improvement in many glucose control parameters in patients with type 1 diabetes during the pandemic (26). The ability of some patients to improve their control may be due to improved diets and exercise regimen that a minority of providers noted in our study.

A finding in our study that may be unique to the US healthcare system is providers noting significant financial stress in patients with diabetes due to job loss and subsequent insurance loss. Early in the pandemic, a report estimated 7.3 million workers in the United States would lose health insurance coverage due to job loss (27). A cross sectional survey conducted April 15-20, 2020 found 28% of respondents had coronavirus related employment or earnings loss. Additionally, 45% of respondents who had COVID job loss are not confident that they could pay for medical care or insurance premiums (28). Though the number of total job losses is less than predicted, it is likely numerous people with diabetes have lost insurance and this has been noted by our participants. There is lack of ongoing research into insurance loss during the COVID-19 pandemic in people with diabetes.

Both primary care and endocrine providers in our study noted the widespread use of telemedicine early in the pandemic with a focus on CGM data review (endocrine specific providers) and increase in lifestyle-based discussions (all clinical team members). Providers did note difficulties with virtual visits in select elderly patients who did not have access to, or knowledge of, specific technologies. The beneficial effects of telemedicine during the COVID-19 pandemic have been noted in studies (29). The perceived and actual benefit to clinicians and patients will need to be an area of further study and development.

Our study included primary care and endocrine providers. Providers reported similar experiences treating patients with diabetes during the early COVID-19 pandemic with a few notable differences. Endocrine providers discussed improvements in self-care in some patients with diabetes and emphasized the benefit of virtual care on CGM and insulin pump monitoring. These differences are likely due to the increased population of patients with type 1 diabetes and use of technology in endocrine specific clinics.

### Strengths/Limitations

To our knowledge, this is the first study to examine clinical team member impressions of challenges facing people with diabetes during the COVID-19 pandemic. Other studies have directly studied patients’ experiences and outcomes with various methods. It is important to study this issue from the clinical team perspective to gain insight into what challenges clinicians perceive to be impacting their patients with diabetes. Additionally, because each clinical team member interacts with multiple patients, they offer a more comprehensive overview of the topic than would be provided by individual patients. Using qualitative methods, we have been able to focus on overarching themes clinical team members have observed during this time.

That said, this study has several limitations. Because we did not directly interview patients with diabetes, our findings are representative of what care team members understood about their patients’ experiences during the pandemic. The comments from providers may not be completely accurate or comprehensive of their patients’ lived experiences. Although our principal interest was to investigate what providers perceived during this time, our overall understanding of the challenges facing people with diabetes during COVID-19 would have been enriched by directly interviewing patients with diabetes. A potential future direction of this work would include interviewing patients to obtain a more complete picture of living with diabetes during COVID-19.

Additionally, our method of purposive sampling has the potential to induce selection bias, as we selected participants based on their ability to provide robust information rather than collecting a random sample. To increase the external validity, we recruited participants from a diverse range of roles and practice settings. However, our study population was limited by high staff turnover and changes in staff roles in clinics due to COVID-19. Though clinical providers were solicited, only a select number responded and participated in the interview process. We were unable to engage every clinical team member, including social workers and mental health workers in clinics who could have added a unique perspective to our findings.

## Conclusion

Clinical team members of patients with diabetes described increases in mental health symptoms, financial stress and disrupted routines leading to self-care challenges during COVID-19. Participants additionally noted the increased use of telemedicine in the care of patients with diabetes during the pandemic. Our study of this clinical team cohort provides information regarding stressors facing patients with diabetes during the pandemic. It is important that these are noted as clinical team members continue to care for patients with diabetes as the pandemic evolves. Mental health support and lifestyle encouragement are critically important. Continued use of telemedicine may help many patients access these services.

## Data Availability Statement

The raw data supporting the conclusions of this article will be made available by the authors, without undue reservation.

## Ethics Statement

The studies involving human participants were reviewed and approved by UNC Chapel Hill Office of Human Research Ethics. Written informed consent for participation was not required for this study in accordance with the national legislation and the institutional requirements.

## Author Contributions

LH: Recruited participants, conducted interviews, coded data, conducted analyses, wrote/edited manuscript. TC: Conducted interviews, coded data, wrote/edited manuscript. MV: Conducted interviews, coded data, conducted analyses, reviewed/edited the manuscript. JL: Developed interview guide, tested codebook, review/edited the manuscript. AJ: Coded data, reviewed/edited the manuscript. KD: Contributed to writing, reviewed/edited the manuscript. ER: Reviewed/edited the manuscript. JR: Study recruitment, reviewed/edited the manuscript. LY: Reviewed, edited manuscript. All authors contributed to the article and approved the submitted version.

## Funding

This work was supported by grant number T35-DK007386 from the National Institutes of Health and by a PCORI Dissemination and Implementation Contract: # DI-2018C1-10853 and NC Translational and Clinical Sciences (NC TraCS) Institute, which is supported by the National Center for Advancing Translational Sciences (NCATS), National Institutes of Health, through Grant Award Number UL1TR002489.

## Conflict of Interest

The authors declare that the research was conducted in the absence of any commercial or financial relationships that could be construed as a potential conflict of interest.

## Publisher’s Note

All claims expressed in this article are solely those of the authors and do not necessarily represent those of their affiliated organizations, or those of the publisher, the editors and the reviewers. Any product that may be evaluated in this article, or claim that may be made by its manufacturer, is not guaranteed or endorsed by the publisher.
